# Double-Loop Hysteresis Behavior and Competing Exchange Interactions in IrMn/NiFe Bilayers

**DOI:** 10.3390/ma19183851

**Published:** 2026-09-10

**Authors:** Byong Sun Chun, Jinseong Jeong

**Affiliations:** 1 Korea Research Institute of Standards and Science, Daejeon 34113, Republic of Korea; mainue@kriss.re.kr; 2 School of Electrical and Computer Engineering, University of Seoul, Seoul 02504, Republic of Korea

**Keywords:** magnetic multilayer, exchange coupling, double loop hysteresis curve

## Abstract

In this work, we investigate the magnetization switching behavior of IrMn/NiFe bilayers as a function of NiFe thickness, temperature, and cooling-field direction. An unusual double-loop hysteresis behavior is observed in IrMn 10 nm/NiFe 40 nm bilayers, whereas a conventional single-loop hysteresis behavior is obtained for thinner NiFe layers. The observed double-loop feature exhibits temperature- and cooling-field-dependent asymmetry, suggesting a spatially nonuniform exchange interaction at the IrMn/NiFe interface. The opposite evolution of switching fields under positive and negative cooling fields indicates competing exchange contributions within the bilayer system. These results are consistent with a model involving distinct interfacial exchange regions or nonuniform uncompensated spin configurations in the IrMn layer. Our findings provide insight into the complex interfacial exchange behavior responsible for double-loop hysteresis in IrMn/NiFe bilayers and highlight the importance of interfacial magnetic inhomogeneity in exchange-biased spintronic structures.

## 1. Introduction

Since the discovery of exchange bias (H_ex_) in 1956 by Meiklejohn and Bean using Co/CoO core–shell particles, it has been extensively studied by numerous research groups [1]. H_ex_ originates primarily from exchange interactions at the interface between antiferromagnetic (AFM) and ferromagnetic (FM) materials, manifesting as a negative or positive shift in the hysteresis loop from the zero-field axis. However, the microscopic origin of exchange bias is not limited to conventional AFM/FM interfacial exchange coupling. Recent studies have demonstrated that exchange bias can also emerge from spin disorder, frustrated magnetic states, and frozen magnetic moments associated with disordered magnetic systems [2,3,4,5]. These findings emphasize that interfacial spin configurations, magnetic disorder, and local magnetic inhomogeneity can play important roles in determining exchange-bias behavior. This phenomenon has received significant attention due to its wide-ranging applications in spintronic devices, such as spin valves and magnetic tunnel junctions. Under an externally applied magnetic field, exchange interactions between the FM and AFM layers generate an effective exchange-bias field, resulting in a shift in the hysteresis loop. However, hysteresis curves occasionally exhibit asymmetric behavior, with a representative example being the double-loop hysteresis curve [6,7,8].

The double-loop hysteresis curve can occur in multilayers consisting of two or more magnetic layers with distinct magnetization properties [9,10]. In such multilayers, when an external magnetic field is applied, the layer with a relatively lower magnetization may reverse its magnetization at lower magnetic fields, whereas the layer with higher magnetization reverses at higher magnetic fields. As a result, two distinct hysteresis loops are observed in the overall magnetization response. In a perpendicularly magnetized magnetic multilayer system, a double-loop hysteresis curve is observed if the magnetization of the FM domain is randomly distributed in two opposite directions and the AFM domain size is larger than or comparable to that of the FM domain [11,12,13]. Additionally, a double-loop hysteresis curve in magnetic tunnel junctions can be caused by defects, such as interfacial roughness in multilayer thin films, oxidation in FM layers, and pinholes in an amorphous tunneling barrier [14,15].

The interface between the AFM and FM layers is affected by various factors, including temperature, the thicknesses of the AFM and FM layers, cooling field strength, and interfacial roughness. Therefore, a clear understanding of its characteristics is of paramount importance. These considerations led us to investigate the exchange coupling of IrMn/NiFe bilayer structures.

The IrMn/NiFe bilayer system is a widely studied AFM/FM platform because IrMn exhibits strong antiferromagnetic ordering and can provide stable exchange bias to NiFe layers. The interfacial magnetic properties of IrMn/NiFe structures are affected by various factors, including AFM and FM thicknesses, cooling-field conditions, temperature, and interface morphology. However, the microscopic origin of unusual switching behaviors, particularly those involving asymmetric or double-loop hysteresis characteristics, remains under active investigation.

In this work, we investigate the magnetization switching behavior of IrMn/NiFe bilayers with different NiFe thicknesses and examine the effects of temperature and cooling-field direction on the hysteresis characteristics. We observe a distinct double-loop hysteresis behavior in the IrMn 10 nm/NiFe 40 nm bilayer, while the IrMn 10 nm/NiFe 20 nm bilayer exhibits a conventional single-loop response. The temperature- and cooling-field-dependent measurements reveal a complex evolution of switching fields and indicate the presence of nonuniform interfacial exchange interactions. Based on these observations, we propose that spatially separated exchange-coupling regions or nonuniform interfacial spin configurations may exist in the IrMn/NiFe bilayer. Unlike many previously reported systems where double-loop hysteresis has been associated with multiple magnetic layers, distinct magnetic configurations, or structural imperfections, the present work suggests that a nonuniform interfacial exchange state in a conventional AFM/FM bilayer can contribute to complex switching behavior. Although the observed behavior is consistent with such an interfacial magnetic inhomogeneity model, direct identification of AFM domain structures requires complementary microscopic measurements beyond conventional magnetometry. This study provides experimental insight into the double-loop hysteresis behavior of IrMn/NiFe bilayers and emphasizes the importance of interfacial exchange variation in determining magnetization reversal processes in a AFM/FM bilayer.

## 2. Experimental Details

Thin-film multilayers with a stacking structure of Si/SiO_2_/Ta 10/IrMn 10/NiFe *t*/Ta 5 nm were fabricated using a six-target DC magnetron sputtering system under a typical base pressure of less than 5 × 10^−8^ Torr. The thickness of the NiFe layer was varied at 20 and 40 nm, whereas the IrMn layer thickness was maintained constant at 10 nm. During the deposition process, an in-plane magnetic field of 100 Oe was applied parallel to the film surface to induce uniaxial magnetic anisotropy in the ferromagnetic NiFe layers. The magnetic properties of the resulting bilayers were subsequently characterized using a Superconducting Quantum Interference Device (SQUID) magnetometer.

## 3. Results and Discussion

**Figure 1:**
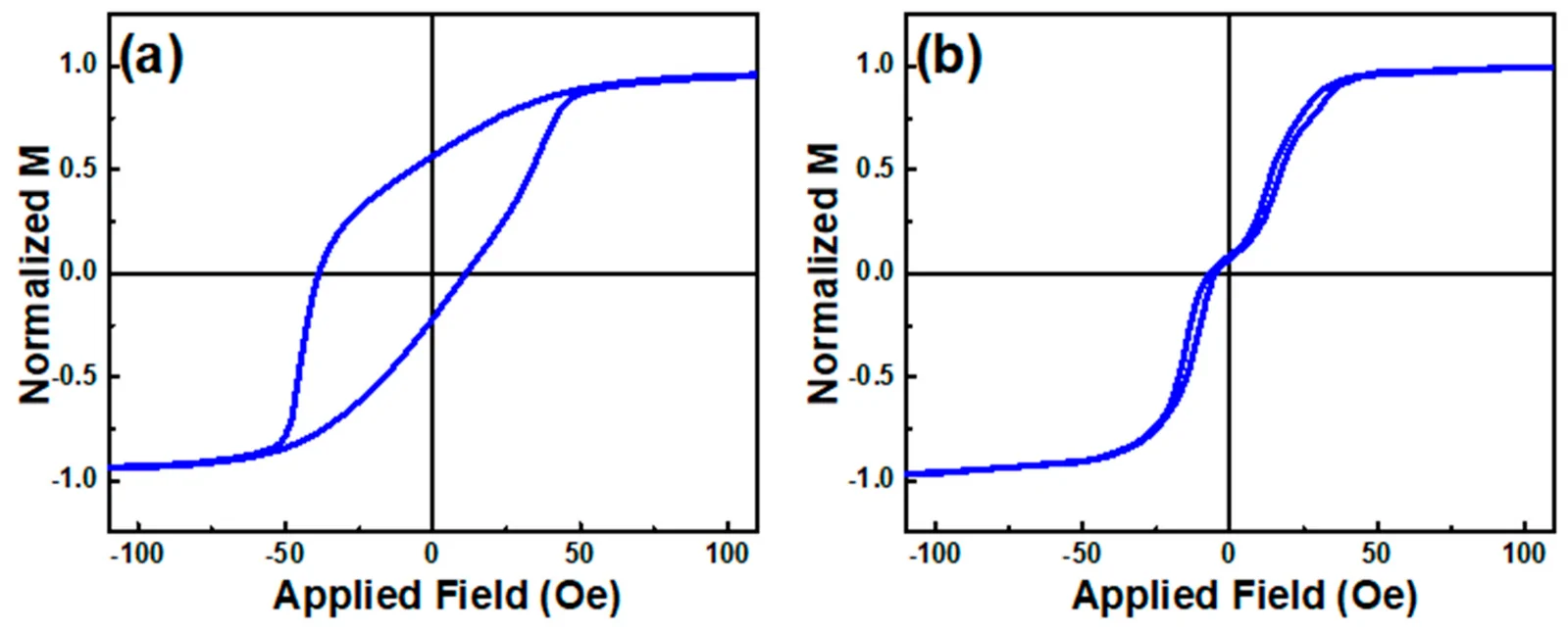
Hysteresis loop of as deposited (**a**) IrMn 10/NiFe 20 nm and (**b**) IrMn 10/NiFe 40 nm.

Figure 1 shows the hysteresis loops of the as-deposited Si/SiO_2_/Ta 10/IrMn 10/NiFe *t*/Ta 5 nm bilayer structures, where the IrMn thickness was kept constant at 10 nm while the NiFe layer thicknesses were varied at (a) 20 nm and (b) 40 nm. The magnetic properties of these two bilayers were measured at room temperature with the external magnetic field applied along the in-plane magnetization direction. The 20 nm thick NiFe film deposited on the 10 nm thick IrMn layer exhibits a single-loop hysteresis curve (Figure 1a). In contrast, the 40 nm thick NiFe film deposited on the same IrMn layer displays a distinctive double-loop hysteresis curve (Figure 1b).

The distinct difference in hysteresis behavior between the 20 nm and 40 nm NiFe samples suggests that the FM layer thickness influences the magnetization reversal process. However, since only two NiFe thicknesses were investigated in this study, further measurements with intermediate thicknesses are required to establish a systematic thickness-dependent evolution of the double-loop hysteresis behavior. Since the IrMn thickness and deposition conditions were identical for both samples, the observed difference is not attributed to a modification of the IrMn layer itself. Although the IrMn layer is deposited before NiFe and the initial AFM domain configuration is therefore established prior to NiFe deposition, the NiFe thickness can influence how the interfacial exchange interactions are reflected during the magnetization reversal process. Increasing the NiFe thickness modifies the balance between interfacial exchange coupling and ferromagnetic volume contributions, which can alter the magnetization reversal pathway and switching behavior of the NiFe layer. Consequently, NiFe regions experiencing different local exchange environments at the IrMn/NiFe interface may undergo magnetization reversal at different magnetic fields, resulting in the emergence of a double-loop hysteresis behavior. Therefore, the role of NiFe thickness is not to generate or define the initial IrMn interfacial spin configuration itself, but rather to regulate the manifestation of spatially varying interfacial exchange interactions during NiFe magnetization reversal.

The double-loop hysteresis behavior was repeatedly observed under identical measurement conditions, whereas the reference IrMn/NiFe structure with 20 nm NiFe exhibited a conventional single-loop hysteresis response. These results suggest that the observed double-loop behavior is intrinsic to the investigated bilayer structure rather than arising from measurement-related artifacts, such as background subtraction errors, sample misalignment, or instrumental effects. Nevertheless, intrinsic magnetic inhomogeneity within the thicker NiFe layer, including possible local variations in anisotropy, strain, microstructure, or magnetic domain structure, cannot be completely excluded. Such intrinsic variations may contribute to complex magnetization reversal behavior in a relatively thick ferromagnetic layer. However, the systematic evolution of the switching fields with temperature and cooling-field direction indicates that interfacial exchange interactions play an important role in determining the observed double-loop hysteresis behavior.

The double-loop hysteresis behavior observed in our system provides insight into the influence of interfacial exchange inhomogeneity on magnetization reversal in conventional AFM/FM bilayers. Unlike several previously reported systems, our structure does not incorporate a perpendicularly magnetized magnetic multilayer, multiple magnetic layers with distinct magnetic properties, or magnetic coupling mediated by pinholes in a tunneling barrier. Therefore, the observed double-loop behavior is unlikely to originate from perpendicular magnetization, magnetic phase separation between multiple ferromagnetic layers, or pinhole-induced coupling effects. Instead, the present results suggest that spatially varying interfacial exchange interactions within a conventional AFM/FM bilayer can generate complex magnetization reversal behavior without requiring additional magnetic layers or artificial coupling structures.

As shown in Figure 1b, the double-loop hysteresis curve exhibits asymmetric magnetization amplitudes relative to the zero axis. The asymmetric magnetization amplitudes indicate that the magnetization reversal process cannot be described by a single homogeneous exchange bias state. Instead, this behavior suggests the coexistence of magnetically distinct regions within the bilayer, where different regions experience different effective exchange fields during magnetization reversal [16]. Furthermore, both subloops of the double-loop curve yield nearly identical coercivity (Hc) and exchange bias field (Hex) values of approximately 13 Oe and 2 Oe, respectively. The comparable switching characteristics of the two subloops further indicate that the observed double-loop behavior originates from multiple exchange-coupled regions rather than from a simple asymmetry in magnetic anisotropy or exchange bias magnitude.

Based on these observations, a possible interpretation is that the IrMn/NiFe interface contains spatially nonuniform exchange-coupling regions. Such regions may originate from variations in uncompensated AFM spins, local anisotropy, grain-dependent exchange interactions, or AFM domain configurations. In this scenario, different NiFe regions coupled to different interfacial spin environments can undergo magnetization reversal at different magnetic fields, resulting in a double-loop hysteresis response.

Although intrinsic magnetic inhomogeneity within the thicker NiFe layer, such as local variations in anisotropy, strain, or microstructure, cannot be completely excluded, the temperature- and cooling-field-dependent evolution of the switching fields suggests that interfacial exchange interactions significantly contribute to the observed double-loop hysteresis behavior.

Although the IrMn layer is deposited before the NiFe layer, the presence of spatially varying interfacial spin configurations in the IrMn layer can influence the subsequent magnetization reversal behavior of the NiFe layer through exchange coupling. Importantly, the NiFe thickness does not determine the formation of these initial IrMn interfacial spin configurations. Instead, the NiFe thickness modifies the magnetic energy balance between interfacial exchange coupling and ferromagnetic volume contributions, thereby affecting the reversal mode and the extent to which the exchange inhomogeneity is manifested in the hysteresis loop. For the thicker NiFe layer, the modified reversal energetics may allow locally different exchange-coupled regions to switch independently, resulting in the experimentally observed double-loop hysteresis behavior.

Therefore, the dependence of the double-loop hysteresis behavior on NiFe thickness should be understood as a change in the manifestation of pre-existing interfacial exchange inhomogeneity rather than a change in the formation of the initial IrMn interfacial spin configuration. This interpretation suggests that spatial variations in IrMn/NiFe interfacial exchange coupling can be selectively revealed depending on the magnetic reversal characteristics of the NiFe layer.

Consequently, the double-loop hysteresis curve observed in our IrMn/NiFe bilayer can be attributed to the presence of spatially nonuniform interfacial exchange regions associated with different IrMn spin configurations. Although this behavior is consistent with the coexistence of oppositely oriented IrMn interfacial domains, direct confirmation of such domain structures requires complementary microscopic characterization techniques. This configuration leading to a discontinuous magnetization loop that manifests as a double-loop hysteresis curve.

**Figure 2:**
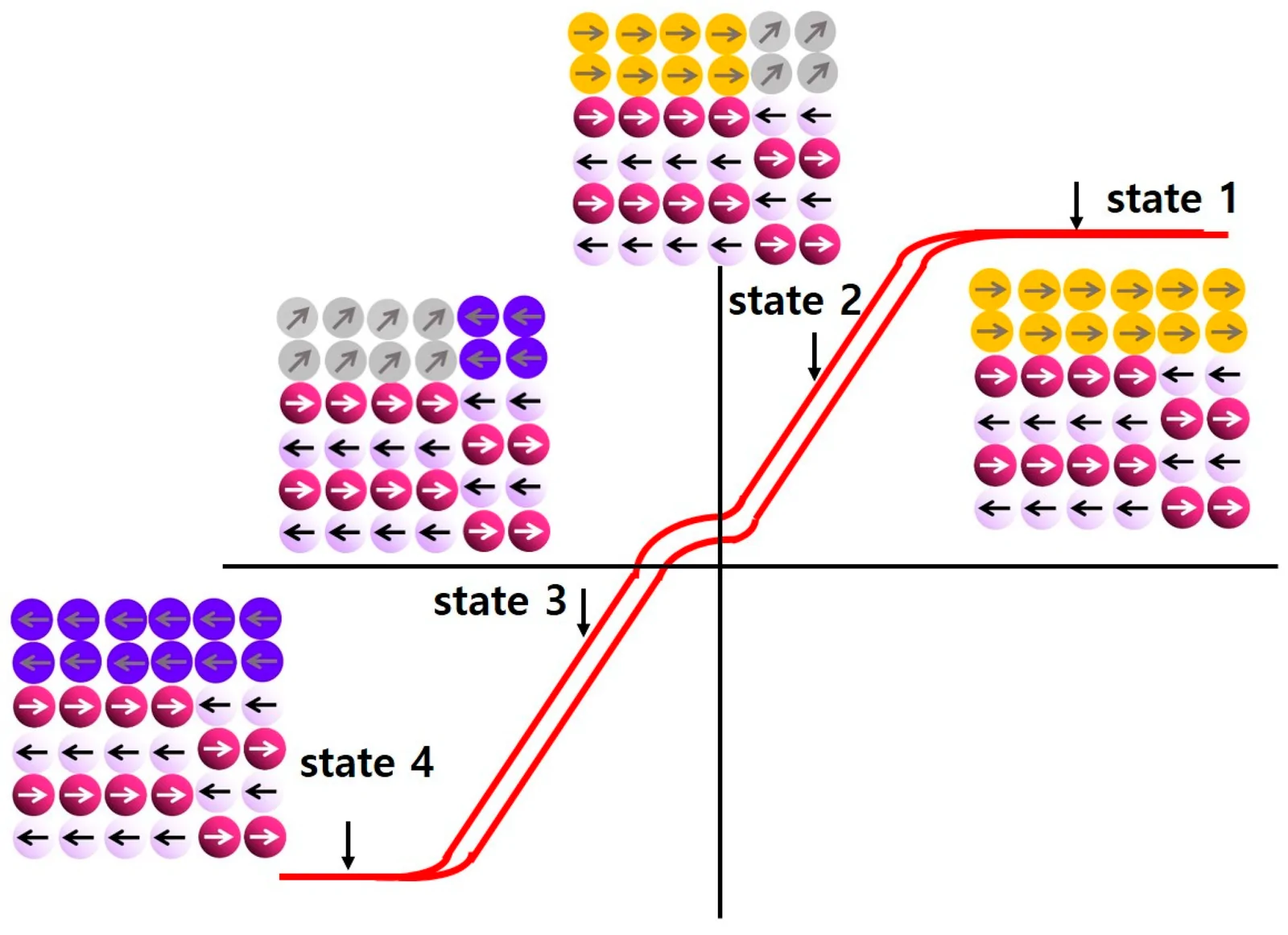
Proposed schematic model illustrating possible spatially nonuniform exchange coupling during magnetization reversal.

To clarify the underlying mechanism of this double-loop hysteresis curve, we propose the following configuration. Figure 2 schematically represents the spin alignment at the interfacial region during the magnetization reversal process. As illustrated, under a high positive external magnetic field, the magnetization directions of the NiFe spins align parallel to the applied field (State 1). Concurrently, two spatially separated bi-domains form in the IrMn layer [12,13,17], with each subsystem possessing interfacial moments oriented either parallel or antiparallel to the external magnetic field direction.

As the external magnetic field decreases, the magnetization of the NiFe layer begins to reverse. However, the magnetization of the NiFe regions locally exchange-coupled with the IrMn layer cannot rotate easily. Instead, the magnetic moments of the NiFe layer under antiferromagnetic exchange coupling with the IrMn layer undergo a slight rotation, which reduces the total magnetization (State 2). Upon a further decrease in the external magnetic field, all magnetic moments of the NiFe layer that are antiferromagnetically coupled to the IrMn align along the -x direction, while the moments in the ferromagnetically exchange-coupled regions rotate slightly (State 3). This further reduces the magnetization into negative values. Finally, when the applied magnetic field increases in the negative direction, all magnetic moments within the NiFe layer align completely along the -x direction (State 4).

Recently, a similar double-loop hysteresis phenomenon was reported in Fe/Cr/Fe/FeCr/Fe/Cr/Fe/FeCr/Fe/Cr/Fe multilayer systems [18]. Specifically, that system exhibited a thermally induced transition characterized by ferromagnetic exchange coupling at high temperatures and antiferromagnetic exchange coupling at low temperatures. Conversely, Fe/Cr/FeCr/Cr/Fe multilayers have been shown to display ferromagnetic exchange coupling at low temperatures and antiferromagnetic exchange coupling at high temperatures.

**Figure 3:**
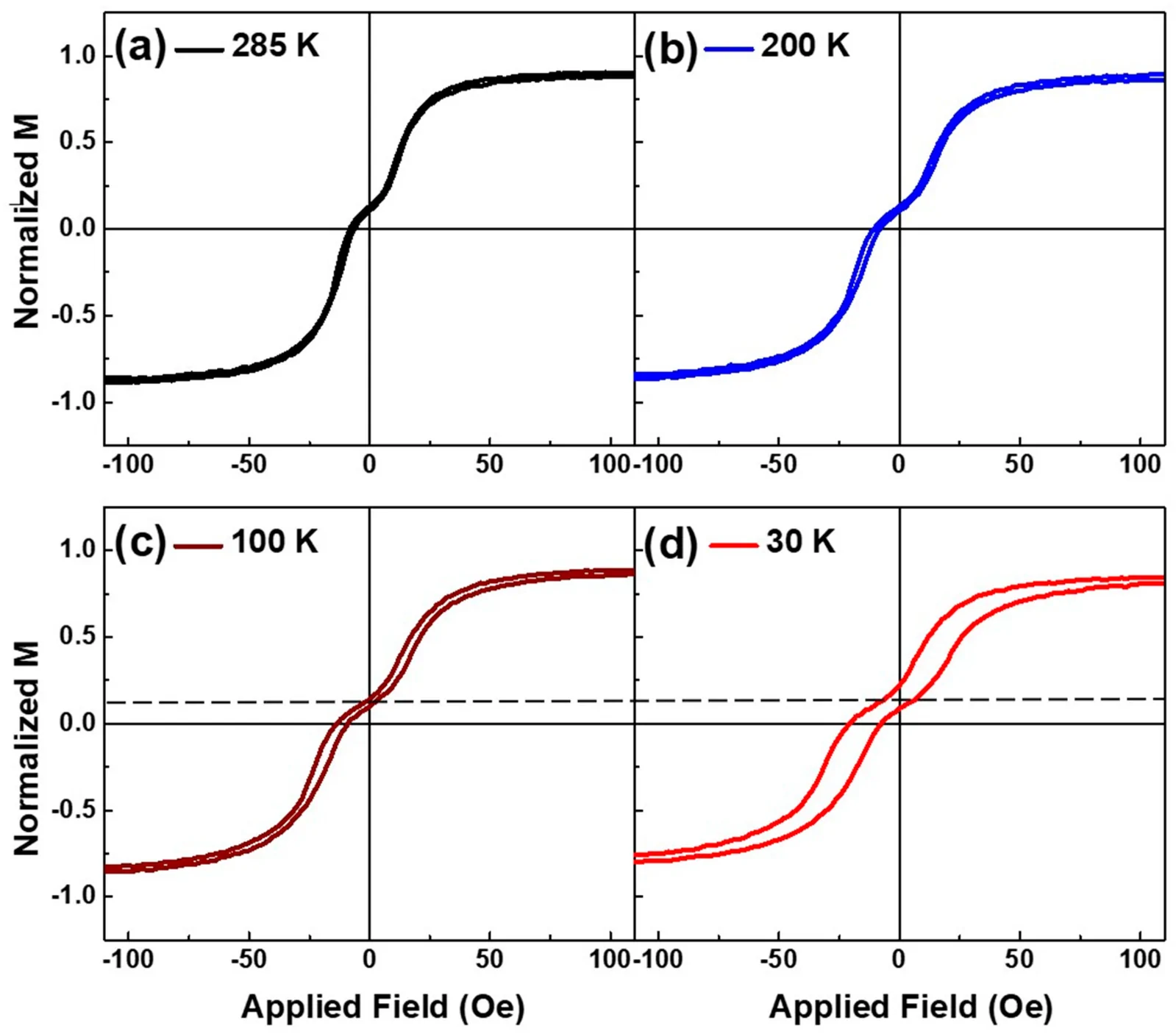
Temperature dependence of the magnetization switching curves. The normalized magnetization value at zero field increases with decreasing temperature.

To elucidate these effects in our system, we investigated the temperature dependence of the exchange coupling. In order to investigate the thermal evolution of the exchange interaction, hysteresis measurements were performed from 285 K to 30 K. Magnetic properties were measured in 50 K increments from 285 K down to 30 K. Representative hysteresis loops obtained at 285 K, 200 K, 100 K, and 30 K are shown in Figure 3.

As shown in this figure, the double-loop feature remains observable over the investigated temperature range, while the normalized magnetization near zero field increases with decreasing temperature. The normalized magnetization value at zero field monotonically increases with decreasing temperature, yielding 0.12 at 285 K, 0.13 at 200 K, 0.14 at 100 K, and 0.22 at 30 K. The persistence of the double-loop feature suggests that the nonuniform exchange configuration is robust over the measured temperature range.

**Figure 4:**
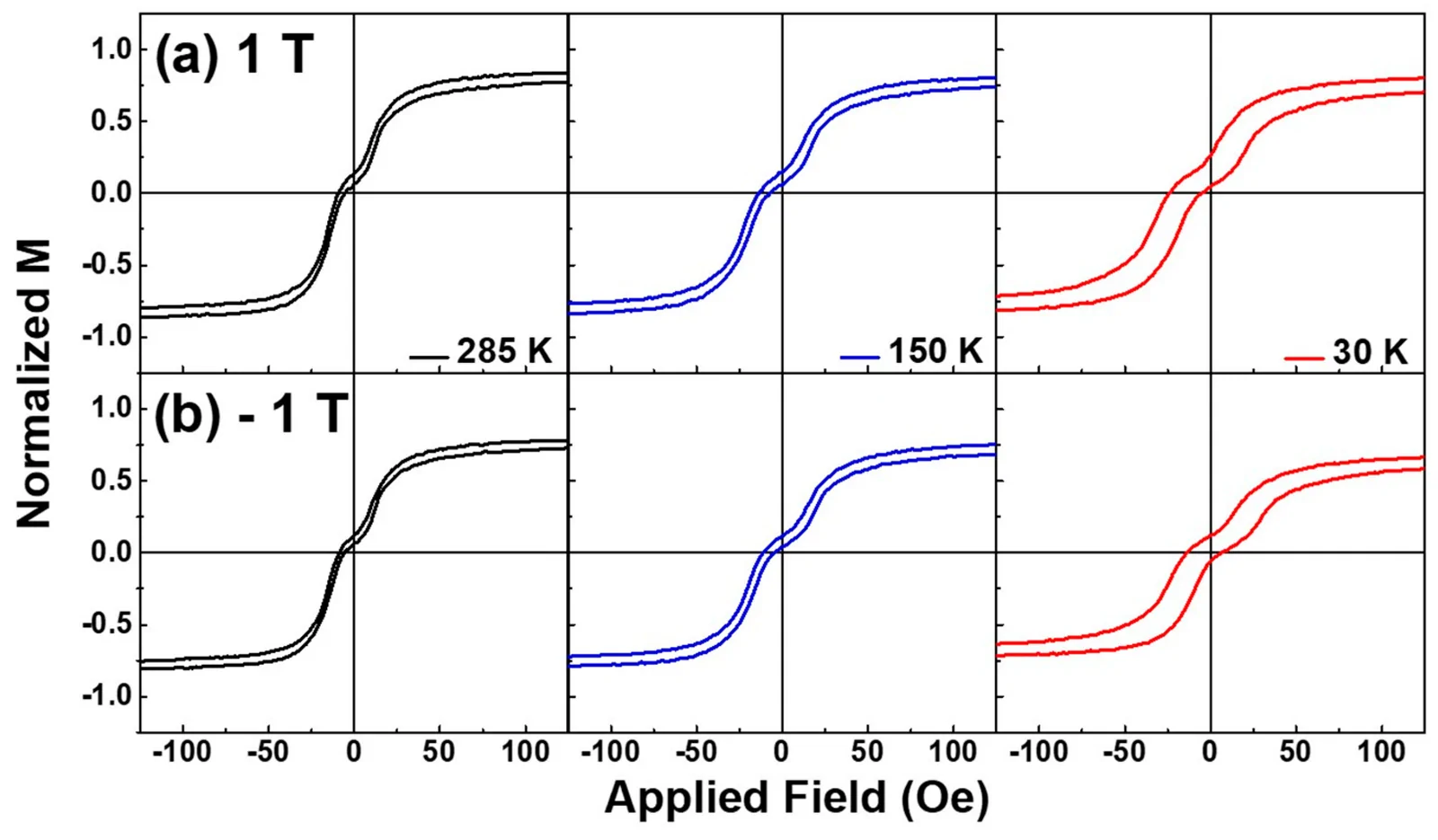
Temperature and cooling field dependence of the magnetization switching curves. (**a**) under a +1 T cooling field and (**b**) under a −1 T cooling field. In both cases, the double loop hysteresis curve shifts in the opposite direction of the cooling field.

We also examined the effects of the cooling field and temperature on the exchange coupling. Figure 4 illustrates the hysteresis loops of the sample measured at 285 K, 150 K, and 30 K under cooling fields of +1 T and −1 T. As shown, the magnetization curves consistently exhibit double-loop profiles under both +1 T (Figure 4a) and −1 T (Figure 4b) cooling fields, closely resembling the behavior observed in the zero-field-cooled state.

Table 1 summarizes the temperature dependence of the external magnetic fields required to reduce the magnetization to zero during field-decreasing (H_L_, descending) and field-increasing (H_R_, ascending) sweeps. We find that the shift in the hysteresis loop increases with decreasing temperature, regardless of the cooling field direction. Notably, the magnetization loop shifts in the direction opposite to the applied cooling field. Under a +1 T cooling field, H_L_ shifts to more negative values as the temperature drops, while H_R_ remains nearly constant. Conversely, under a −1 T cooling field, H_L_ maintains an approximately constant value with decreasing temperature, whereas the negative magnitude of H_R_ decreases, eventually becoming positive below 50 K.

The hysteresis loops measured after cooling under +1 T and −1 T fields exhibit systematic changes in the switching-field positions. The loop displacement depends on the cooling-field direction, indicating that the interfacial spin configuration is sensitive to the direction of the cooling field. The opposite evolution of the switching fields under positive and negative cooling fields suggests that the exchange configuration at the IrMn/NiFe interface is influenced by the cooling-field direction. These results indicate the presence of competing exchange contributions rather than a single uniform exchange-bias state. Although the cooling-field-dependent measurements demonstrate the sensitivity of the interfacial exchange configuration to the cooling-field direction, a systematic investigation using various cooling-field magnitudes and direct observation of AFM domain structures would be necessary to quantitatively determine the evolution and population of the proposed interfacial spin configurations. Therefore, the present results support the existence of spatially varying exchange interactions at the IrMn/NiFe interface, while the exact microscopic origin of these exchange configurations remains to be further clarified.

In a system dominated by antiferromagnetic coupling at the AFM/FM interface, the magnetic moments of the two constituent materials align antiparallel to each other. When the FM material is exposed to a cooling field, its magnetic moments align along the field direction, forcing the AFM interfacial moments to align in the opposite direction due to the antiferromagnetic coupling. This configuration causes the resulting hysteresis curve to shift opposite to the cooling field direction. In contrast, when ferromagnetic exchange coupling occurs between the FM and AFM layers, the system resides in a low-energy state where the exchange coupling direction remains unaltered by the cooling field. Therefore, the opposing directional shifts in the hysteresis loops (shifting negative at +1 T and positive at −1 T) depending on the cooling field direction confirm that the IrMn layer hosts two spatially separated dual domains, each possessing interfacial moments oriented either parallel or antiparallel to the external magnetic field.

## 4. Conclusions

In conclusion, we observed double-loop hysteresis behavior in an IrMn/NiFe bilayer. The emergence of the double-loop response with increasing NiFe thickness indicates that the ferromagnetic layer thickness plays a critical role in determining the interfacial exchange configuration. The asymmetric magnetization amplitudes and comparable coercivity and exchange-bias fields of the two subloops suggest the presence of magnetically distinct regions with different effective exchange interactions. Temperature-dependent and field-cooling-dependent measurements further reveal that the double-loop behavior originates from a spatially nonuniform interfacial exchange state. We propose that spatially nonuniform IrMn interfacial spin configurations induce locally different exchange coupling environments, resulting in sequential magnetization reversal processes within the NiFe layer. Further systematic studies involving IrMn thickness variation are necessary to elucidate the evolution of AFM domain configurations and their relationship with double-loop hysteresis behavior. This work provides new insight into interfacial spin inhomogeneity in exchange-biased systems and demonstrates that complex magnetization reversal behavior can be achieved through engineering of AFM/FM interfaces.

## Figures and Tables

**Table 1:** Temperature dependence of the magnitude of the external magnetic field required to bring the magnetization to zero.

		285 K	250 K	200 K	150 K	100 K	50 K	30 K
+1 T	H_L_/H_R_	−9/−5	−10/−6	−12/−7	−14/−7	−15/−8	−20/−7	−24/−7
−1 T	H_L_/H_R_	−9/−6	−9/−5	−10/−5	−11/−5	−11/−3	−12/1	−12/6

## Data Availability

The original contributions presented in this study are included in the article. Further inquiries can be directed to the corresponding author.

## Abbreviations

The following abbreviations are used in this manuscript:

H_ex_	Exchange Bias
AFM	Antiferromagnetic
FM	Ferromagnetic
SQUID	Superconducting Quantum Interference Device
